# Sleep apnoea in patients undergoing colorectal cancer surgery: prospective cohort study

**DOI:** 10.1093/bjsopen/zrag025

**Published:** 2026-04-15

**Authors:** Martin Claesson, Eva Lindberg, Carin Sahlin, Anders Blomberg, Markku M Haapamäki, Malin Sund, Johan Svensson, Jenny Theorell-Haglöw, Malin Jonsson Fagerlund, Karl A Franklin

**Affiliations:** Department of Diagnostics and Intervention, Surgery, Umeå University, Umeå, Sweden; Department of Medical Sciences, Respiratory, Allergy and Sleep Research, Uppsala University, Uppsala, Sweden; Department of Public Health and Clinical Medicine, Umeå University, Umeå, Sweden; Department of Public Health and Clinical Medicine, Umeå University, Umeå, Sweden; Department of Diagnostics and Intervention, Surgery, Umeå University, Umeå, Sweden; Department of Diagnostics and Intervention, Surgery, Umeå University, Umeå, Sweden; Department of Surgery, University of Helsinki and Helsinki University Hospital, Helsinki, Finland; Department of Diagnostics and Intervention, Surgery, Umeå University, Umeå, Sweden; Department of Statistics, Umeå School of Business, Economics and Statistics, Umeå University, Umeå, Sweden; Department of Medical Sciences, Respiratory, Allergy and Sleep Research, Uppsala University, Uppsala, Sweden; Perioperative Medicine and Intensive Care and Department of Physiology and Pharmacology, Karolinska University Hospital and Karolinska Institutet, Stockholm, Sweden; Department of Diagnostics and Intervention, Surgery, Umeå University, Umeå, Sweden

## Abstract

**Background:**

Patients with sleep apnoea are at increased risk of postoperative cardiac and respiratory complications, but its prevalence in patients undergoing abdominal cancer surgery is poorly defined. The aim of this study was to estimate the prevalence of sleep apnoea in patients undergoing colorectal cancer surgery compared with community-based controls, and to assess the utility of symptoms, signs, and the STOP-Bang questionnaire in identifying sleep apnoea.

**Methods:**

This prospective observational study included consecutive patients scheduled for colorectal cancer surgery at Umeå University Hospital, Sweden, between 7 January 2015 and 24 May 2020. Overnight polysomnography, including electroencephalogram-based sleep scoring, was undertaken before operation. Prevalence estimates were compared with those of two community-based cohorts from Uppsala, Sweden (female participants from the Sleep and Health in Women cohort; male participants from the Men in Uppsala: A Study of Sleep Apnoea and Cardiometabolic Health cohort). The primary outcome was the prevalence of sleep apnoea.

**Results:**

Of 268 eligible patients, 5 were receiving continuous positive airway pressure therapy and 201 underwent successful polysomnography. Moderate-to-severe sleep apnoea was identified in 98 of 206 patients (48 (95% confidence interval 41 to 54)%) undergoing colorectal cancer surgery, with a similar prevalence in men and women. Among 597 community-based controls, moderate-to-severe sleep apnoea was present in 27 (24 to 31)%. After adjustment for age, sex, and body mass index, the odds ratio for moderate-to-severe sleep apnoea in patients with colorectal cancer was 1.57 (95% confidence interval 1.08 to 2.29; *P* = 0.019). The STOP-Bang questionnaire demonstrated acceptable sensitivity (85 (95% confidence interval 76 to 91)%) but low specificity (42 (33 to 52)%).

**Conclusion:**

Approximately half of patients undergoing colorectal cancer surgery have moderate-to-severe sleep apnoea, exceeding the prevalence in community-based controls. Clinical symptoms, signs, and STOP-Bang screening are insufficient to reliably identify affected patients, indicating that overnight sleep apnoea assessment is required in this population.

## Teaser

This study shows that moderate-to-severe sleep apnoea is overrepresented and affects half of patients undergoing colorectal cancer surgery. Overnight sleep apnoea investigations are necessary to determine whether a patient undergoing colorectal cancer surgery have sleep apnoea or not.

## Introduction

Sleep apnoea is a common disease but most patients with this condition are undiagnosed when undergoing surgery^1^. These individuals are at increased risk of postoperative complications, including cardiovascular events, hypoxia, respiratory failure, and unexpected admissions to intensive care units^2–8^. Clinical guidelines^9,10^ recommend assessing patients for symptoms and signs of sleep apnoea and use of the STOP-Bang questionnaire^11^ to identify patients at risk before sleep apnoea investigations, and perioperative continuous positive airway pressure (CPAP) therapy in severe cases^12^.

At many centres, patients undergoing bariatric surgery are screened routinely for sleep apnoea and managed with perioperative CPAP therapy to mitigate postoperative complications^13,14^. Sleep apnoea is seldom recognized in patients undergoing elective cancer surgery. Although treatable, only one study^15^ was identified that investigated the prevalence of sleep apnoea in 37 patients undergoing abdominal surgery. There is a significant knowledge gap regarding the prevalence of sleep apnoea in patients undergoing abdominal cancer surgery.

The aim of this study was to assess the prevalence of sleep apnoea among patients undergoing elective colorectal cancer surgery compared with that in community-based controls. A secondary objective was to assesses the utility of symptoms and signs including STOP-Bang in identifying patients with sleep apnoea.

## Methods

### Ethical statement

The Regional Ethical Review Board at Umeå University approved the study to investigate patients with colorectal cancer (2012–375-31 M) and controls from senior citizen organizations (2016-181-32). The Regional Ethical Review Board at Uppsala University approved the Sleep and Health in Women study (2011/244) and Men in Uppsala: A Study of Sleep, Apnoea and Cardiometabolic Health study (2016/029). All participants provided written informed consent, and the studies adhered to the Declaration of Helsinki. This study is reported in accordance with the STROBE statement for cohort studies. A completed STROBE checklist is provided in the *Supplementary material*.

### Outcomes

The main outcome was the prevalence of sleep apnoea assessed using overnight polysomnography in a cohort of consecutive patients scheduled for colorectal cancer surgery at Umeå University Hospital in Sweden. This prevalence was compared with the prevalence of sleep apnoea in two community-based cohorts, with adjustments for age, body mass index (BMI), sex, hypertension, and current smoking.

In patients with colorectal cancer, secondary outcomes were the value of single symptoms and signs, and the STOP-Bang questionnaire based on symptoms and signs in identifying sleep apnoea. Other secondary outcomes included cardiovascular postoperative complications, and severe complications (> IIIb according to the Clavien–Dindo classification)^16^.

### Patients with colorectal cancer

Consecutive patients with colorectal cancer scheduled for surgery on Tuesdays and Wednesdays at the Department of Surgery, Umeå University Hospital, were included from 7 January 2015 to 24 May 2020. Patients with cognitive impairment were excluded. Patients already receiving nocturnal CPAP therapy because of sleep apnoea continued with this treatment before and after surgery, and were therefore excluded from polysomnography. All patients followed an enhanced recovery after surgery protocol, and all had clinical follow-up at 30 days after surgery.

### Community-based population controls

**Fig. 1:**
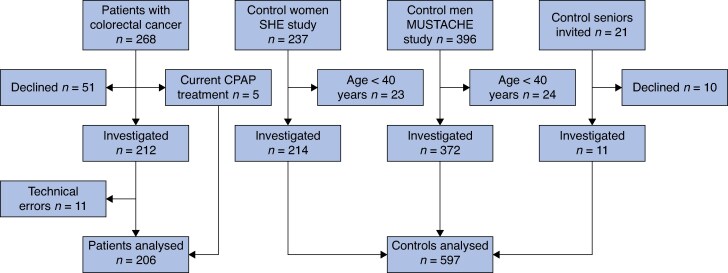
Study flow chart SHE, Sleep and Health in Women; MUSTACHE, Men in Uppsala: A Study of Sleep Apnoea and Cardiometabolic Health; CPAP, continuous positive airway pressure.

The controls were participants in two community-based cohort studies conducted in Uppsala, Sweden. Female controls were recruited from the Sleep and Health in Women cohort that includes 237 randomly chosen women from the general population^17^. Male controls were recruited from the Men in Uppsala: A Study of Sleep, Apnoea and Cardiometabolic Health cohort. This cohort comprises 396 men recruited from the population-based Epi-Health study (341) and by means of local advertisements (59)^18^. Some 24 men and 23 women aged < 40 years were excluded from the control cohorts to better match their age to patients with colorectal cancer, and an additional 21 men and women aged > 70 years were also invited from a local senior organization in Umeå, Sweden (*Fig. 1*).

### Measurements

Polysomnographic recordings (Embla Titanium, Flaga, Iceland) were performed in hospital the night before surgery in patients with colorectal cancer, and at home in controls from the population. The recordings included the continuous recording of electroencephalograms (EEGs) (C3, C4, O1, O2, F3, F4 in patients with colorectal cancer; C3, C4, Fz in female controls; and C3, C4, O2, Fz in male controls), electro-oculograms, submental electromyograms, airflow with a nasal flow pressure sensor, respiratory effort from piezoelectric belts, finger pulse oximetry, electrocardiograms (V5), and body position sensors.

The same certified polysomnographic scorer (C.S.) manually scored all the recordings according to the American Academy of Sleep Medicine^19^ rules in both patients with colorectal cancer and controls. Obstructive apnoea was defined as a flow reduction of ≥ 90% for at least 10 seconds (s) with continuing abdominal and thoracic movements. Obstructive hypopnoea was defined by a 30% decrease in nasal pressure compared with baseline for at least 10 s, accompanied by ongoing abdominal and thoracic movements, along with arousal or ≥ 3% drop in oxygen saturation. Central apnoea was scored as the cessation of nasal pressure for 10 s without respiratory movements. Sleep was scored in 30-s epochs. The apnoea–hypopnoea index (AHI) was calculated as the mean number of apnoeas and hypopnoeas per hour of sleep. Mild, moderate, and severe sleep apnoea were defined by an AHI value of ≥ 5 to < 15, 15 to < 30, and ≥ 30, respectively. The oxygen desaturation index represented the mean number of oxygen desaturations ≥ 3% per hour of sleep. Nocturnal hypoxia was measured as the time in minutes of oxygen saturation < 90% during sleep.

Patients with colorectal cancer answered questions on witnessed sleep apnoeas and snoring while asleep. The response options were always, often, sometimes, seldom, never, and I don’t know^17,20^. Witnessed apnoeas were considered at a reported frequency of always, often or sometimes. Habitual snoring was considered if snoring frequency was rated as always or often. A score of ≥ 10 on the Epworth sleepiness scale indicated excessive daytime sleepiness. The Mallampati classification was used as a proxy for tongue size^21^. The STOP-Bang tool was used to estimate the risk of sleep apnoea^11^. Height and weight were measured the day before polysomnography.

### Statistical analysis

To ensure sufficient statistical precision, a sample size of 200 patients with colorectal cancer was calculated, allowing for a 95% confidence interval with a 7% margin of error around an estimated proportion of 50%. Clopper–Pearson confidence intervals were used for estimating confidence intervals for proportions. A logistic regression analysis, adjusted for sex, age group (≤ 60, 61–70, 71–80, and ≥ 81 years), BMI, hypertension, and current smoking was undertaken to compare the prevalence and severity of sleep apnoea between patients and community-based controls. A sensitivity analysis using propensity score matching with respect to age group, sex, BMI, hypertension, and current smoking was also carried out because of the different age distribution in patient and control cohorts. The Kruskal–Wallis test, with Bonferroni correction for multiple comparisons, was used to compare the time with nocturnal hypoxia and severity of sleep apnoea. Prediction precisions were quantified by means of sensitivity and specificity with 95% confidence intervals.

A significance level of 0.05 was selected. SPSS^®^ version 28.0 was used for data analysis. Graphs were prepared using GraphPad Prism^®^ 9.4.1 for MacOS^®^.

## Results

### Characteristics of patients with colorectal cancer and controls

A total of 268 patients with colorectal cancer were invited to participate. Of these, 51 declined. Five men were treated with CPAP, four with severe and one with moderate sleep apnoea. They used CPAP before and after surgery, and were not investigated with polysomnography. In all, 212 patients underwent polysomnographic recordings, with a further 11 excluded because of technical errors (*Fig. 1*). The sample comprised 206 patients with colorectal cancer, 201 with successful recordings and 5 on CPAP; there were 68 women and 138 men, with a mean(standard deviation) age of 70(10) years and a mean BMI of 26.7(4.0) kg/m^2^. Patients who declined to participate (51 individuals) were more often women (82 *versus* 35%; *P* < 0.001), and had slightly lower BMI (24.8 *versus* 26.7 kg/m^2^; *P* = 0.013) compared with those included for polysomnography (212). There was no difference in mean age (70.5 years in both groups; *P* = 0.97).

Mallampati grade and time with oxygen saturation < 90% were missing for eight patients with colorectal cancer and STOP-Bang scores for three patients. A sleeping pill was taken by five patients, and one patient took pain medication before polysomnography.

The control group comprised 597 individuals with complete polysomnographic recordings (*Fig. 1*). They were 223 women and 374 men, with a mean age of 61(10) years and mean BMI of 26.2(4.4) kg/m^2^.

### Primary outcome—prevalence of sleep apnoea

**Fig. 2:**
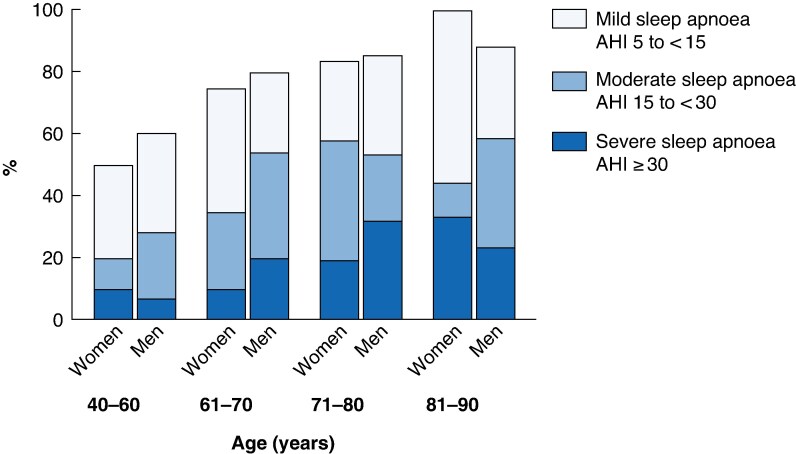
Prevalence and severity of sleep apnoea in women and men with colorectal cancer AHI, apnoea–hypopnoea index.

Among the investigated patients with colorectal cancer, the prevalence of mild-to-severe sleep apnoea was 79 (95% confidence interval (c.i.) 74 to 85)%. The prevalence of moderate-to-severe sleep apnoea was 48 (41 to 54)%, with no difference between women and men (44 (32 to 56) *versus* 49 (41 to 58)%, respectively; *P* = 0.50) (*Fig. 2*). Sleep apnoea was classified as mild in 32 (25 to 38)% of patients, moderate in 27 (21 to 33)%, and severe in 21 (16 to 27)%.

Characteristics of patients with colorectal cancer with and without sleep apnoea are shown in *Table 1*. There was a weak, but statistically significant correlation between age and AHI (*r* = 0.244, *r*^2^ = 0.059, *P* < 0.001), and between BMI and AHI (*r* = 0.250, *r*^2^ = 0.062, *P* < 0.001). The mean AHI was 13.3 (95% c.i. 10.5 to 16.0) among patients aged < 70 years, which was significantly lower than the value of 21.4 (18.4 to 24.4) among those aged ≥ 70 years (*P* < 0.001). Eighteen patients (9%), 15 men and 3 women, had a previous diagnosis of sleep apnoea. Of these, 16 patients had moderate-to-severe sleep apnoea and 2 had mild sleep apnoea. Five patients were using CPAP, whereas none of the remaining 13 patients had any ongoing treatment.

**Table 1:** Characteristics of patients with colorectal cancer with and without sleep apnoea

	Sleep apnoea			*P*†
	None, AHI < 5 (*n* = 43)	Mild, AHI 5–15 (*n* = 65)	Moderate-to-severe, AHI > 15 (*n* = 98)	
Age (years), mean(s.d.)	65(11)	70(10)	72(9)	0.001‡
BMI (kg/m^2^), mean(s.d.)	25.5(3.2)	26.2(4.1)	27.6(4.2)	0.008‡
**Sex**				0.779
Female	15 (35%)	24 (37%)	29 (30%)	
Male	28 (65%)	41 (63%)	67 (68%)	
Neck circumference (cm), mean(s.d.)	39(3)	39(4)	40(4)	0.004‡
Witnessed sleep apnoea	4 (9%)	11 (17%)	17 (19%)	0.377
Snoring	6 (14%)	12 (19%)	25 (28%)	0.165
Daytime sleepiness*	16 (37%)	31 (49%)	42 (46%)	0.498
Hypertension	17 (40%)	35 (54%)	61(62%)	0.044
Diabetes mellitus	4 (9%)	3 (5%)	17 (19%)	0.022

Values are *n* (%) unless otherwise stated. *Epworth sleepiness scale ≥ 10. AHI, apnoea–hypopnoea index; s.d., standard deviation; BMI, body mass index. †? test, except ‡? test.

**Fig. 3:**
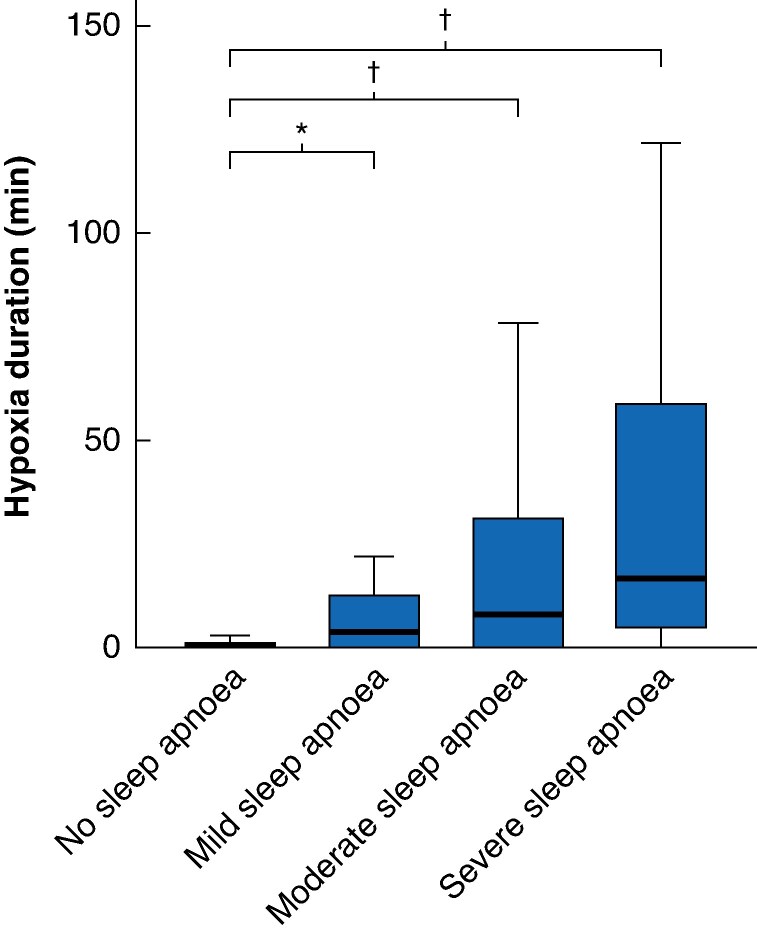
Sleep apnoea and nocturnal hypoxia among patients with colorectal cancer Duration of oxygen saturation < 90% during sleep according to severity of sleep apnoea. Bold horizontal lines, boxes, and error bars represent median, interquartile range, and range, respectively. min, Minutes. *P = 0.001, †*P* < 0.001 (Kruskal–Wallis test, with Bonferroni correction).

In patients with colorectal cancer, the mean oxygen desaturation index was 19.2 (95% c.i. 17.2 to 21.3), and the mean duration of nocturnal hypoxia was 7.3 (95% c.i. 5.4 to 9.1)% of total sleep time. The occurrence of nocturnal hypoxia was more prevalent in patients with sleep apnoea, and this prevalence increased with the severity of the condition (*Fig. 3*).

Among the 597 community-based controls, the prevalence of sleep apnoea was 55 (51 to 59)%, and moderate-to-severe sleep apnoea occurred in 27 (24 to 31)%, with no significant difference between women and men (25 (19 to 31) *versus* 29 (24 to 34)%, respectively; *P* = 0.289). There was a significant correlation between age and AHI (*r* = 0.324, *r*^2^ = 0.105, *P* < 0.001), and between BMI and AHI (*r* = 0.352, *r*^2^ = 0.124, *P* < 0.001). The mean AHI was 9.7 (8.6 to 10.9) among community-based controls aged < 70 years compared with 18.3 (15.2 to 21.3) for those aged ≥ 70 years (*P* < 0.001).

**Fig. 4:**
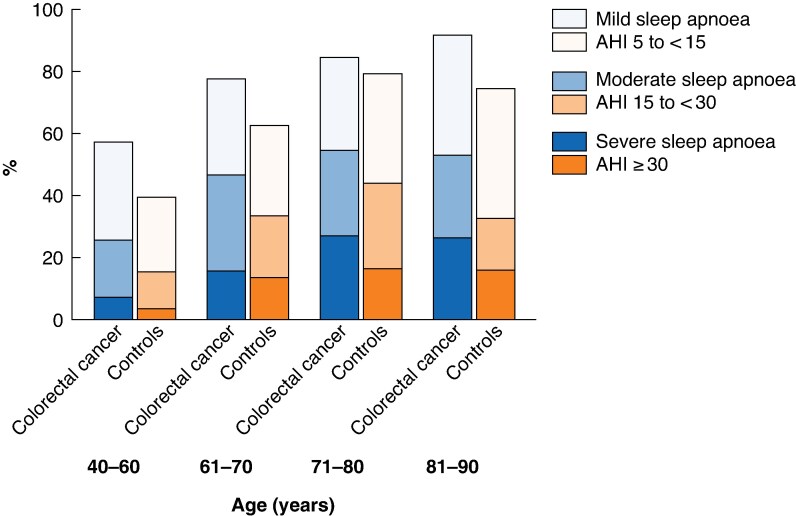
Prevalence of sleep apnoea in patients with colorectal cancer and controls Sleep apnoea was more common in patients with colorectal cancer than the control population: adjusted odds ratio 1.71 (95% confidence interval 1.11 to 2.62; *P* = 0.015). AHI, apnoea–hypopnoea index.

Sleep apnoea was more prevalent in patients with colorectal cancer than in controls (*P* < 0.001) (*Fig. 4* and *Table 2*). The crude odds ratio (OR) for mild-to-severe sleep apnoea was 3.10 (95% c.i. 2.14 to 4.51; *P* < 0.001) in patients with colorectal cancer compared with controls. After adjusting for BMI, sex, age group, smoking and hypertension, the adjusted OR was 1.71 (1.11 to 2.62; *P* = 0.015). The adjusted OR for moderate-to-severe sleep apnoea was 1.57 (1.08 to 2.29; *P* = 0.019). In propensity score-matched sensitivity analysis, the OR for moderate-to-severe sleep apnoea was 1.54 (1.07 to 2.21; *P* = 0.022).

**Table 2:** Prevalence of sleep apnoea in patients with colorectal cancer and community-based controls

	Patients with colorectal cancer (*n* = 206)	Controls (*n* = 597)	P*
Age (years), mean(s.d.)	70(10)	61(10)	< 0.001†
BMI (kg/m^2^), mean(s.d.)	26.7(4.0)	26.2(4.4)	0.149†
**Sex**			0.387
Female	70 (34%)	221 (37%)	
Male	136 (66%)	376 (63%)	
Current smoker	2 (1%)	34 (5.7%)	< 0.001
Hypertension	113 (54%)	159 (27%)	< 0.001
Sleep apnoea, AHI > 5	163 (79%)	328 (55%)	< 0.001
Moderate-to-severe sleep apnoea, AHI > 15	98 (48%)	162 (27%)	< 0.001

Values are *n* (%) unless otherwise stated. s.d., Standard deviation; BMI, body mass index; AHI, apnoea–hypopnoea index. *? test, except †? test.

### Secondary outcomes in patients undergoing colorectal cancer surgery

The mean sleep duration was 323 (95% c.i. 307 to 340) minutes, with 16 (95% c.i. 15 to 17)% spent in stage 1 sleep, 53 (50 to 55)% in stage 2, 17 (16 to 18)% in stages 3 and 4, and 14 (13 to 16)% in rapid eye movement sleep. The sleep efficiency was 73 (95% c.i. 70 to 76)%.

The value of symptoms and signs in identifying sleep apnoea before surgery was low (*Table 3*). Sleep apnoea was more prevalent than symptoms and signs resulting in a low sensitivity for BMI ≥ 30 kg/m^2^, neck circumference, habitual snoring, witnessed sleep apnoea, daytime sleepiness with Epworth sleepiness scale ≥ 10, and Mallampati grade. The sensitivity for STOP-Bang in identifying moderate-to-severe sleep apnoea was 85%, but as many as 139 of 198 patients (70%) had a STOP-Bang score of ≥ 3 and the specificity was merely 42%.

**Table 3:** Sensitivity and specificity of symptoms and signs in identifying sleep apnoea before colorectal cancer surgery

	Frequency	Sleep apnoea*		Moderate-to-severe sleep apnoea†	
		Sensitivity (%)	Specificity (%)	Sensitivity (%)	Specificity (%)
BMI ≥ 30 kg/m^2^	38 of 201	22 (15, 29)	91 (78, 97)	23 (15, 32)	84 (76, 91)
Neck circumference ≥ 40 cm	73 of 198	41 (33, 49)	77 (61, 88)	46 (36, 57)	71 (61, 79)
Habitual snoring	26 of 201	14 (9, 20)	91 (78, 97)	14 (8, 23)	88 (80, 93)
Witnessed sleep apnoea	30 of 201	18 (12, 25)	95 (84, 99)	17 (10, 26)	87 (79, 93)
Epworth sleepiness scale ≥ 10	15 of 199	7 (4, 12)	91 (78, 97)	7 (2, 14)	92 (85, 96)
Mallampati grade ≥ 3	24 of 193	11 (7, 17)	83 (67, 93)	10 (5, 18)	86 (77, 92)
STOP-Bang score ≥ 3	139 of 198	75 (68, 82)	49 (33, 65)	85 (76, 91)	42 (33, 52)

Values in parentheses are 95% confidence intervals. *Apnoea–hypopnoea index (AHI) ≥ 5; †AHI ≥ 15. BMI, body mass index.

Cardiovascular complications occurred within 30 days of surgery in 10.8% of patients with moderate-to-severe sleep apnoea (AHI ≥ 15), compared with 5.4% of patients with AHI < 15 (*P* = 0.17). Some 11% of patients had serious complications, with a Clavien–Dindo grade of ≥ IIIb after colorectal cancer surgery. There was no significant difference in rate of serious complications between patients with moderate-to-severe sleep apnoea and without sleep apnoea. Two patients died within 30 days of surgery, and both had moderate-to-severe sleep apnoea. One was an 85-year-old man with an AHI of 54 who died from respiratory failure after a right hemicolectomy. The other, a 76-year-old man with an AHI of 22, succumbed to anastomotic insufficiency and respiratory failure after a left hemicolectomy. None of the five men with CPAP-treated sleep apnoea experienced any complications. As they received treatment both before and after operation, they were not classified as having sleep apnoea in the analysis of complications.

## Discussion

This study demonstrated a high prevalence of sleep apnoea in patients undergoing colorectal cancer surgery. Some 79% of patients were diagnosed with sleep apnoea, with 48% presenting moderate-to-severe disease, which occurred equally in men and women. Nocturnal hypoxia was associated with the severity of the condition. Given this unexpectedly high prevalence of sleep apnoea, a comparative analysis was conducted using a community-based control group assessed with similar equipment and interpretation criteria. The prevalence of sleep apnoea was higher among patients with colorectal cancer than in community-based controls; this was partly attributable to older age, but the association remained after adjusting for age, sex, BMI, smoking, and hypertension. Symptoms and clinical signs of sleep apnoea showed low sensitivity in identifying patients with sleep apnoea before surgery. Although the STOP-Bang questionnaire demonstrated higher sensitivity, its specificity was low.

Sleep apnoea increases with age^17,22,23^, and age is one explanation for the high prevalence of sleep apnoea among patients undergoing colorectal cancer surgery. It is known that sleep apnoea is common in patients undergoing cardiac and bariatric surgery, and many patients are investigated and treated for this condition before bariatric surgery. However, few have been investigated before abdominal surgery. Roggenbach *et al*.^15^ investigated 37 patients undergoing major abdominal surgery using simplified sleep apnoea recordings. Of these, 38% had mild sleep apnoea and 22% had moderate-to-severe disease. The prevalence increased substantially in the postoperative period, and sleep apnoea in the postoperative phase was not restricted to patients with this condition before surgery. They also report that snoring and daytime sleepiness were unable to identify patients with sleep apnoea.

Current sleep apnoea guidelines, contrary to the present findings, recommend identifying patients with sleep apnoea based on symptoms and signs before obtaining a preoperative sleep apnoea recording^9,10,13^. The low values of symptoms and signs can explain why surgeons and anaesthetists fail to identify patients with sleep apnoea before surgery^1^. The present results indicated that habitual snoring, reporting witnessed sleep apnoea, and daytime sleepiness had a low sensitivity, and many patients with sleep apnoea were overlooked. Many patients with sleep apnoea in this study were asymptomatic, explaining why single symptoms and signs had a low sensitivity in identifying sleep apnoea in patients with colorectal cancer. STOP-Bang, which includes a combined symptom and sign score, had better sensitivity. However, the majority of patients (139 of 198, 70%) had a STOP-Bang score of ≥ 3, and the sensitivity for moderate-to-severe sleep apnoea was still no better than 85%, and the specificity was only 42%. The present results demonstrated that an overnight sleep study is necessary to determine whether a patient undergoing colorectal cancer surgery has sleep apnoea.

In the present study, overnight polysomnography with EEG-based sleep staging was conducted in a hospital setting. This represents the standard level 1 diagnostic modality and is well suited for research purposes. However, its complexity and cost render it impractical for routine preoperative screening. A simplified home sleep apnoea test is more feasible in clinical practice, particularly level 3 sleep studies incorporating respiratory parameters and heart rate monitoring^24^. Although level 3 investigations are generally preferable, a previous study indicated that overnight pulse oximetry alone (level 4) increases the validity of STOP-Bang^25^.

A limitation of the present study is that it lacked sufficient power to determine whether sleep apnoea was associated with postoperative complications. A *post hoc* power calculation suggested that 870 patients would be required, assuming a 100% relative increase in the risk of cardiovascular complications among individuals with moderate-to-severe sleep apnoea (10.8 *versus* 5.4%), a prevalence of moderate-to-severe sleep apnoea of 50%, a power of 80%, and a significance level below 0.05. Likewise, 3584 patients would be needed to detect a 30% relative increase in the risk of severe complications (Clavien–Dindo ≥ IIIb) among those with moderate-to-severe sleep apnoea (13 *versus* 10%). A weakness of the study is the age difference between patients with colorectal cancer and controls. Although this issue was mitigated through adjusted analyses and a propensity score-matched sensitivity analysis, a more robust approach would have been to compare patients with colorectal cancer with age-matched controls from the outset. Patients underwent polysomnography in hospital on the night before surgery, a setting that may have adversely affected sleep quality and thereby the occurrence of sleep apnoea events. Nevertheless, total sleep time and the distribution across sleep stages were close to age-related norms, supporting the reliability of the investigations^26^.

Despite a relatively low mean BMI of 26 kg/m², this study demonstrated that almost half of both men and women undergoing colorectal cancer surgery have moderate-to-severe sleep apnoea and nocturnal hypoxia that warrant CPAP therapy. Nevertheless, only five patients (2.4%), all men, were receiving CPAP treatment. These findings underscore the extent to which sleep apnoea remains undetected, despite its well established association with increased risks of cardiovascular disease, and possibly also with cancer^27,28^. Importantly, neither clinical signs nor the STOP-Bang questionnaire successfully identified patients with sleep apnoea before surgery.

The strongest evidence linking sleep apnoea to postoperative complications comes from a study by Chan *et al*.^8^, which investigated 1218 patients with co-morbid disease undergoing major non-cardiac surgery. It was found that patients with previously unrecognized severe sleep apnoea had an increased risk of cardiovascular complications, with an adjusted hazard ratio of 2.2 (95% c.i. 1.5 to 3.3). CPAP therapy has been reported to improve oxygenation, and reduce the need for reintubation and mechanical ventilation after surgery in patients with sleep apnoea^12^. Given that approximately half of patients undergoing abdominal cancer surgery in the present cohort met criteria for CPAP treatment, there is a need for large, multicentre prospective studies evaluating postoperative outcomes in this population, as well as trials assessing whether perioperative CPAP reduces complication rates.

Patients with severe sleep apnoea and co-morbid diseases are at increased risk of postoperative cardiac complications^8^. Rather than limiting evaluation to individuals who present with symptoms or clinical signs of sleep apnoea, the authors propose that patients with co-morbid disease (American Society of Anesthesiologists grade III–IV) should undergo routine preoperative assessment for sleep apnoea.

## Supplementary Material

zrag025_Supplementary_Data

## Data Availability

Deidentified data underlying this article will be shared on reasonable request. Requests should be directed to the corresponding author.
